# Eating Disorders in Relationship with Dietary Habits among Pharmacy Students in Romania

**DOI:** 10.3390/pharmacy6030097

**Published:** 2018-09-01

**Authors:** Magdalena Iorga, Isabela Manole, Lavinia Pop, Iulia-Diana Muraru, Florin-Dumitru Petrariu

**Affiliations:** 1 Department of Behavioral Sciences, Faculty of Medicine, University of Medicine and Pharmacy “Grigore T. Popa” of Iasi, Iasi 700115, Romania; magdalena.iorga@umfiasi.ro; 2 Faculty of Pharmacy, University of Medicine and Pharmacy “Grigore T. Popa” of Iasi, Iasi 700115, Romania; isabella0290@gmail.com; 3 Nutrition and Dietetics, Faculty of Medicine, University of Medicine and Pharmacy “Grigore T. Popa” of Iasi, Iasi 700117, Romania; daianapopovici85@yahoo.com; 4 Department of Preventive Medicine and Interdisciplinarity, Faculty of Medicine, University of Medicine and Pharmacy “Grigore T. Popa” of Iasi, Iasi 700115, Romania; fpetrariu@mail.com

**Keywords:** pharmacy student, diet habits, eating disorders, anorexia, bulimia, BMI

## Abstract

Changing dietary habits of university students is due to personal, social, educational or religious factors. The relationship between dietary habits and presence of eating disorders among university students is less known in Romania. **Material and Methods**: Ninety-one pharmacy students (91.21% women) were included in the research. Socio-demographic, anthropometric, medical, and psychological data were collected. Dietary self-declared habits were registered. The analysis of data was done using SPSS, v23. **Results**: A total of 69.2% of students had normal weight, 64.84% preferred to have lunch, and 23.08% eat during nights. The majority of subjects (95.6%), stated that they eat snacks daily. More than one-third of students keep diets to reduce their weight. Younger students tend to eat more main meals per week, snack more, and eat later after getting up in the morning. Subjects with high body dissatisfaction tended to have fewer main meals (r = −0.265, *p* = 0.011) and to skip breakfasts (−0.235, *p* = 0.025) and dinners (r = −0.303, *p* < 0.001). Pharmacy students that presented higher rate of emotional problems tend to sleep less and skip breakfast. **Conclusions**: Female pharmacy students had higher mean scores on all subscales than those found among Romanian women. A strong relationship between dietary habits and eating disorders was identified.

## 1. Introduction

During their university studies, students change their dietary habits, and several factors were found to be responsible for it: changing location, living arrangements (skipping home-prepared food), respecting school schedule (leading to an increased number of snacks and skipping meals), having an easy access to fast-food (university campuses are providing fast-food for students), self-administrating palatable meals (students have the tendency to eat preferred meals), limitation by costs and easy to find food, etc. [1,2,3,4,5].

The persistence of dietary habits is not limited in time. These dietary habits were found to be stable over the years that is why developing a healthy nutritional behavior during academic years is important for both physical and mental health. Students were considered as a vulnerable group because they fail to meet dietary requirements for a long period of time. Lower scores for stress, bad eating behaviors, or poorer dietary habits were registered among students compared to general population. Studies revealed that medical students are practicing less healthy behaviors or eat more unhealthy food compared to non-medical students [6,7].

Unhealthy food is associated with high rates of chronicity, like obesity and cardiovascular disease, both identified in general population and among students. Some studies pointed that there was an increased rate of obesity among university students in the last twenty years; students start to gain weight since freshmen years and this process slows but still increases during the adult life [8,9,10]. Obesity and overweight rates registered among medical students were closely related to some dietary behaviors like: skipping breakfast, frequent fast food and low consumption of fruits and vegetables, or easy access to unhealthy food and carbohydrates from food machines or convenient fast-food stores [11,12,13,14,15].

Apart from these reasons, eating habits are also related to socio-cultural reasons. Western cultures are more prone to accept and promote thinness compared to eastern countries. Studies pointed out the influence of media on the ideal weight and how that ideal models influence to self-image of the body and also that the ideal weight of the body is closely related to the cultural ideal model. That is why, cultures where thinness is more agreed are more prone to registered high rates of eating disorders like anorexia and bulimia.

Acculturative stress is another aspect related to changing dietary habits for students who are studying abroad. One of the stressors is represented by the impossibility to find their favorite meals, their individual preferred food, or ingredients that they are used to using. Dietary acculturation is an extra distressful factor that may affect a student’s life [16,17,18].

There is no other study in Romania investigating the relationship between eating disorders and dietary habits among pharmacy students. The aim of the study was to evaluate the presence of eating disorders among Romanian pharmacy students and their relationship with the patterns of dietary habits.

## 2. Materials and Methods

### 2.1. Study Design and Participants

A total of 91 students were included in the present research. All participants were enrolled, at the time of the investigation, in “University of Medicine and Pharmacy “Grigore T. Popa” of Iasi, Romania, Faculty of Pharmacy, from all five years of study. In total, the number of pharmacy students registered was 700, so the subjects investigated represented almost 10% of the targeted population.

The students voluntarily participated to the research. Participants were informed about the purpose of the study and they were informed from the beginning that they could withdraw from the study anytime they wanted to, with no penalties. The survey took approximately 25 min to complete.

A total of 120 questionnaires were distributed directly by the investigators. From the 115 returned documents (with a rate of response of 96%), 91 were taken into consideration for the research. Willingness to participate was considered as an inclusion criterion. The exclusion criteria were: documents were not fully filled-in, or were returned after the requested dead-line.

### 2.2. Questionnaires

A combined questionnaire including socio-demographic, medical, anthropometric, and psychological data was created.

#### 2.2.1. Socio-Demographic Data

Because the present study is the first one focusing on eating habits and disease among pharmacy students in Romania, information like age, gender (male/female), environment (rural/urban), and year of study (one to five) were registered for this cross-sectional study.

#### 2.2.2. Anthropometric and Medical Data

Information like weight, weight, body mass indices (BMI) and the existence of a chronic disease were also registered. Height and weight data were converted into Quetelet’s BDI and the value was considered using *World Health Organization* (WHO) standards for European population: a BMI < 18.5 kg/m^2^ was categorized as underweight, 18.5–24.9 kg/m^2^ as the normal range, 25.0–29.9 kg/m^2^ as pre-obese, 30–34.9 kg/m^2^ as obese class I, 35.0–39.9 kg/m^2^ as obese class II, and ≥40 kg/m^2^ as obese Class III [10].

Also, an item was addressed in order to identify if students were satisfied with their weight, to declare if they used diets in order to reduce their weight. Information about having a chronic disease or being under medical treatment were also gathered.

#### 2.2.3. Dietary Data

The study focused also on gathering information regarding the consumption of vegetables, fruits, snacks, or fast-food. The selection of dietary data was collected in order to respect also the religion and the restriction imposed by the practice of it.

#### 2.2.4. Health-Related Behaviors

Special items were constructed in order to identify sleep-related problems (the number of hours of sleep), the number of weekly breakfasts, lunches, or dinners, preferred meal, serving snacks, the tendency to skip meals, eating during the night, and fasting.

#### 2.2.5. Eating Disorders

Psychological problems referring to eating disorders were evaluated using *Eating Disorder Inventory* (*EDI*). The original tool was designed by Gamer [19] for identifying eating disorders, and it is widely used both in research and in clinical practice to screen symptoms and psychological features related to eating disorder.

For the present study, EDI-3, Romanian Form [20] was used. The 91 items investigate the following aspects: three scales are related specific to eating disorders (drive for thinness—DT, bulimia—B, and body dissatisfaction—BD) and nine are general psychological scales in strong relationship with eating disorders (low self-esteem—LSE, personal alienation—PA, interpersonal insecurity—II, interpersonal alienation—IA, interoceptive deficits—ID, emotional dysregulation—ED, perfectionism—P, ascetism—AS, and maturity fears—MF).

A 6-point scale represents the response options for the items of EDI-3, ranging from *always* to *never*. The instrument has a number of 25 reversed items. In this case, we also reverse the coding. It is mandatory to choose a response for each item and individuals have to decide which one suits those best. The score for each subscale is obtained by summing up all scores for that scale.

EDI-3 is not an inventory designed for diagnosing eating disorders, but a tool to assess symptoms relevant to the development and maintaining of eating disorders. High scores on the first three scales are related to high risks of developing eating disorders. The other nine scales take into consideration important psychological aspects relevant to the evolution and persistence of eating disorders.

The adaptation and validation of EDI-3 on the Romanian population used individuals from the general population from six of the 41 counties in Romania and individuals with a diagnosis of eating disorders. For most of the subscale, there were no significant differences between male and female participants (in accordance with other studies), with two exceptions: drive for thinness and body dissatisfaction, with women scoring higher than men on these scales.

Test-retest reliability indicates a high stability in time for EDI-3, correlation coefficients varying between 0.613 and 0.844, with *p* < 0.001. The instrument also shows a good internal consistency on all scales as measured by alpha Cronbach coefficients ranged from 0.600 to 0.899. Correlations between the scores on the scales of EDI-3 and other eating disorders relevant instruments (Eating Attitudes Test-26, Rosenberg self-esteem scale, Eysenck Personality Questionnaire, Beck Depression Inventory, Endler Multidimensional Anxiety Scales, etc.) indicate a good criterion validity. Intercorrelations between the scales of EDI-3 and factor analysis point to a good construct validity.

### 2.3. Statistical Analysis

The statistical analysis was done using SPSS IBM, version 23. (IBM, Tokio, Japan). Descriptive analysis used mean and standard deviations; Independent Samples *t* Tests were used in the case of one independent, categorical variable that has two levels/groups and one continuous dependent variable. The one-way analysis of variance (ANOVA) was also used to establish whether there are any statistically significant differences between the means of three or more independent (unrelated) groups. For Pearson correlations, interval measured variables were used. Differences were considered statistically significant at P value <0.05.

## 3. Results

### 3.1. Socio-Demographic, Anthropometric, and Medical Data

The majority of participants were females (N = 83, 91.21%), age range of 18–39 (M = 22.30 ± 2.71). The majority of female students is a common feature, the rate being specific to all rates registered by medical universities.

Socio-demographic (gender, age, environment), anthropometric (weight and BMI), and medical (having a chronic disease or being under medical treatment) data are described in Table 1.

### 3.2. Psychological Data—EDI-3

Results for EDI-3 subscales are presented in Table 2. The results obtained for both genders are presenting in Table 2, together with scores for men and women from general population in Romania. Comparing with women from general population, scores for female pharmacy students are higher for all subscales. For men, only five scores were registered to be higher than that of Romanian males: drive for thinness, body dissatisfaction, personal alienation, interoceptive deficits, and emotional dyssregulation.

There is no association between age and BMI (r = 0.059, *p* = 0.582). Pearson correlations revealed negative significant associations between age and number of main meals per week (r = −0.265, *p* = 0.012) and the number of snacks per day (r = −0.244, *p* = 0.021). Also, there is a positive correlation between age and eating after getting up in the morning (r = 0.337, *p* = 0.001). More specifically, younger students tend to eat more main meals per week, snack more, and eat later after getting up in the morning.

Pearson correlations revealed that students with a high drive for thinness have higher body weights (r = 0.282, *p* < 0.001) and tend to skip dinners (r = −0.344, *p* < 0.001). As expected, a positive correlation was identified between weight and the subscales body dissatisfaction (r = 0.342, *p* < 0.001) and drive for thinness (r = −0.285, *p* < 0.001). Also, participants with high body dissatisfaction tend to have fewer main meals (r = −0.265, *p* = 0.011) and to skip breakfasts (r = −0.235, *p* = 0.025) and dinners (r = −0.303, *p* < 0.001).

Interpersonal alienation (reluctance to form close relationship) and maturity fears (the fear to face the demands specific to an adult life) had a negative correlation with number of lunches per week (r = −0.295, *p* < 0.001 and r = −0.214, *p* = 0.042, respectively), meaning that participants with high scores on this subscale tend to skip lunch.

For the subscale interoceptive deficits, it was identified a positive correlation with the number of eaten fruits (r = 0.252, *p* = 0.021). Interoceptive deficits scale measures the ability of an individual to discriminate between sensations (of hunger and satiety) and feelings.

A negative correlation was identified between emotional dysregulation and the number of hours of night sleep and number of breakfasts (r = −0.222, *p* = 0.035). So, pharmacy students that presented higher rate of emotional problems tend to sleep less and skip breakfast.

Also, a negative correlation was identified between ascetism and the number of main meals per week (r = −0.265, *p* = 0.012), meaning that students with high scores on ascetism tend to skip more main meals.

### 3.3. Diatery Habits and Health-Related Behaviors

#### 3.3.1. Sleep-Related Data

Students were asked to estimate the number of nightsleep hours. The results revealed an M = 7 ± 1.02 h of sleep every night and almost a quarter of them (24.12%) were having a nap after lunch almost every day.

The *t* test revealed significant differences between subjects who eat during nights (M = 11.85) and those who do not (M = 9.54) on personal alienation (t(89) = 2.31, *p* = 0.035). Personal alienation refers to low self-esteem. Results proved that students who used to eat during nights had higher scores on personal alienation.

#### 3.3.2. Meals, Snacks, and Diets

Students were asked to mention which was their **preferred meal**: 64.84% of them usually prefer have lunch, 23.08% dinner, and 12.09% enjoy having breakfasts. A number of 21 subjects (23.08%) declared that they eat during nights. They had also to mentions how many times they succeed in serving breakfasts, lunches, and dinners. Results are presented in Table 3.

Students were also asked to report whether they eat snacks or not. The majority of them (95.6%), sustained that they eat snacks daily, with an M = 2.33 ± 1.25.

More than one-third of students declared that they used to keep diets to reduce their weight (N = 37, 40.7%). When comparing students who dieted for reducing weight to those who did not, the first group obtained statistically significant higher scores on each of the following six subscales: drive for thinness (t(89) = 6.14, *p* < 0.001), bulimia (t(89) = 3.03, *p* = 0.004), body dissatisfaction (t(89) = 3.71, *p* < 0.001), personal alienation (t(89) = 2.01, *p* = 0.047), emotional dysregulation (t(89) = 2.02, *p* = 0.046), and ascetism (t(89) = 3.25, *p* = 0.002).

#### 3.3.3. Religious Fasts and Vegetable/Fruit Diet

The results of the Independent Samples *t* Test revealed that students who fast (M = 9.24) had lower scores than those who do not (M = 12.96) on interpersonal alienation (t(89) = −3.21, *p* = 0.002).

The answers to the items regarding the daily consumption of fruits and vegetables proved that 35.2% of students eat fruits daily and 57.1% of them declared they eat daily vegetables, with an M= 2.02 ± 1.41 fruits and M = 5.92 ± 1.54 vegetables per day.

One-Way ANOVA analyses showed significant differences between fruit consumption (1—daily, 2—two times a week, 3—three, four times a week, 4—not at all) and three of the subscales: drive for thinness (F(3.87) = 4.57, *p* = 0.005), emotional dysregulation (F(3.87) = 3.24, *p* = 0.026), and perfectionism (F(3.87) = 3.86, *p* = 0.012). The multiple comparisons analysis showed that students who ate fruits daily (M = 11.53) had a higher drive for thinness than those who eat fruits twice a week (M = 5.23); those who do not eat fruits (M = 14.80) had higher scores on emotional dysregulation than those who eat fruits three/four times a week (M = 6.00); and participants who do not eat fruits (M = 16.60) had higher scores on perfectionism than those who used to eat fruits two times (M = 9.52) or three/four times a week (M = 9.97).

#### 3.3.4. Satisfaction with Personal Weight

A number of 53 (58.2%) students declared that they are content with their weight. The results of the Independent Samples *t* Tests showed significant differences between subjects who are content with their weight and those who are not on 9 of the 12 subscales of the EDI-3: drive for thinness (t(89) = −5.40, *p* < 0.001), bulimia (t(89) = −4.42, *p* < 0.001), body dissatisfaction (t(89) = −8.31, *p* < 0.001), low self-esteem (t(89) = −3.08, *p* = 0.003), personal alienation (t(89) = −4.36, *p* < 0.001), interpersonal alienation (t(89) = −3.21, *p* = 0.002), interoceptive deficits (t(89) = −3.78, *p* < 0.001), emotional dysregulation (t(89) = −3.47, *p* = 0.001), and ascetism (t(89) = −4.85, *p* < 0.001). More specifically, students who are content with their weight have a lower drive for thinness, lower scores on bulimia, higher self-esteem, lower scores on personal and interpersonal alienation, lower interoceptive deficits and emotional dysregulation, lower scores on ascetism, and are more satisfied with their bodies comparative with students dissatisfied with their weight.

#### 3.3.5. Environment

Environment is closely link to several aspects of nutrition (more healthy food, private gardeners with fruits and vegetables) and more religious people. The analysis of data focused on the relationship between environment and the consumption of food (vegetables, fruits) or respecting religious fasts.

The chi square test revealed no statistical difference between students from rural areas and those from urban areas according to whether they respect religious fasts or not (χ^2^(2) = 3.716, *p* = 0.054) or whether they eat fruits (χ^2^(3) = 1.173, *p* = 0.759) or vegetables (χ2(1) = 0.004, *p* = 0.951) on a daily basis.

Regarding EDI-3 analysis of data, the Independent Samples *t* Test, reveled a significant difference between students from **rural** areas (M = 15.59) and those from urban areas (M = 11.70) concerning the interpersonal insecurity (II) subscale of the EDI-3 (t(89) = −2.91, *p* = 0.004). More specifically, students from rural area score higher on interpersonal insecurity (II) than those from urban areas.

#### 3.3.6. BMI

One-way ANOVA was used in order to identify the statistically significant differences between BMI categories. Significant differences were identified between underweight (M = 3.92), normal weight (M = 8.74), and pre-obese (M = 13.71) participants regarding the subscale DT (F(2.85) = 5.43, *p* = 0.006). Multiple comparisons using the Bonferroni method showed a significant difference between underweight and pre-obese students, the first group having a lower drive for thinness than the last group.

Significant statistical differences were also identified between underweight (M = 11.38), normal weight (M = 15.11), and pre-obese (M = 25.08) participants concerning the body dissatisfaction (BD) subscale (F(2.85) = 6.98, *p* = 0.002). Underweight students had lower scores on body dissatisfaction than pre-obese participants, the latter obtained higher scores than the former.

Positive correlations were identified between BMI and drive for thinness (r = 0.291, *p* < 0.001), bulimia, (r = 0.391, *p* < 0.001), body dissatisfaction (r = 0.447, *p* < 0.001), and ascetism (r = 0.246, *p* = 0.019).

## 4. Discussion

The present study identified a weight of M = 60.2 ± 10.56 (ranged from 42 to 105 kg) and 69% of students are normal weight. No obese persons were identified among the questioned students. Because no other study was lead on pharmacy students’ weight-related aspects, no comparative analysis could be done considering other results on Romanian pharmacy students. A previous study lead on Romanian students from many specialties, developed in 2012, registered a lower M = 54.3 ± 4.7 for investigated Romanian student population. At that time, the score was lower in comparison with German students, with an M = 60.3 ± 9.3 [21].

For female participants, our sample had higher mean score on all subscales than those found among Romanian women. For men, the majority of mean scores were higher than those reported among men from Romania, with the exception of low self-esteem, interpersonal insecurity, interpersonal alienation, ascetism, and maturity fears, where the means were lower [20].

A study lead on Hungarian medical and pharmacy students showed a low administration of milk, fruits, and vegetables [22]. Similar results were found by Allen et al. focusing on Canadian pharmacy students, identifying that students’ dietary habits were far below Canadians’ Food Guide recommendations [23]. Interesting results targeting students in Egypt showed that there is an important effect of nutrition awareness and knowledge on health habits and performance among Egyptian pharmacy students, meaning that knowledge is not sufficient to stimulate students from Pharmacy to practice healthy habits, this must be doubled by nutrition awareness [24]. Complementary results were presented by Tiralongo and Wallis, who showed that Australian pharmacy students must internalize first information prior respecting nutritional alternatives [25].

Sleep has consequences on both physical and psychological health. The results regarding the duration of night sleep showed that questioned students had an M = 7 ± 1.02 h of sleep every night. *American Academy of Sleep Medicine* recommended for this category of age 7–9 h for an optimal health [26].

The studies lead on pharmacist students in USA [27], Malaysia [28], and Poland [29] or focusing on working pharmacists [30] revealed that there is a high rate of consumption of vitamins, minerals, or other supplements and rates are even higher compared to other medical specialties or other science studies. It is natural to think that students or health professionals who are well-trained in drugs and their effect on health to consume with precociousness these medications [27]. One raison for high rate for the consumption of supplements could be related to the fact that these medical specialists are trained to be more aware about the importance of nutritional status and they are able to evaluate the fact that skipping meals or consuming unhealthy food lead to an unbalanced nutritional status with negative consequences on health. This self-evaluation of their dietary behaviors doubled by the knowledge in drug’s component could be a reason for a self-administration of medicines. This habit was identified among medical students by a lot of researches focusing on this population [31,32] and among working pharmacists, with or without presenting a chronic disease [33,34]. The rates of self-administration of dietary supplements among pharmacy students balanced in different studies between 20% in Japan [35] and 47% in USA [27].

Our study identified that over 40% of the students admitted to dieting to lose weight, a higher rate than those observed in other studies [36]. Our results concerning the positive correlation between three scales of the EDI-3 (specifically the drive for thinness, bulimia, and body dissatisfaction) and weight and BMI are in accordance with those found in the Romanian population, with the exception of the relationship between bulimia and weight, where we found no associations [20].

The results pointed out that higher BMIs are associated with higher score on body dissatisfaction. Similar results found by Jaworowska and Bazylak [37]. The authors identified a relatively low percentage of pharmacy students satisfied with their body weights (34.4% for female and 37.1% for male participants), despite the fact that they were not overweight or obese. As-Sa’edi et al. [38] also found, a low percentage of female medical students satisfied with their bodies (26.40%), while the rest perceived themselves as either to thin or too heavy and expressed a desire to lose weight. In our study, only students with high BMIs tend to have a higher drive for thinness.

Female students desire a significantly thinner figure than men [39] and that their ideal figure is underweight [6]. In our study, a high desire to be thin is usually associated with high BMI. Albertson et al. [40] showed that a self-compassion meditation training spanning a period of 3 weeks improved body satisfaction for women.

Another finding in our sample is that students with high BMIs tend to score high on the bulimia scale of EDI-3, suggesting that this group of students are at a higher risk of developing symptoms for bulimia. Findings from other studies [41] point to the fact that weight suppression has an important effect on bulimic symptoms and this association could be maintained by the preoccupation with thinness.

When comparing college students and eating disorder patients, findings showed that the latter have lower mean levels of self-esteem [42] and that students with greater positive body image have higher levels of self-esteem [43]. Research suggest that, when comparing individuals with eating disorders with individuals without eating disorders, the first group has a tendency towards perfectionism in maladaptive ways [44]. Our results that students who do not eat fruits have higher scores on perfectionism than those who eat fruits two times or three, four times a week, suggesting that perfectionism could have significant implications for health and well-being.

Also, the results showed that that students with high scores on ascetism tend to skip more main meals. The results are in concordance with Gamer’s results [19]. The ascetic motive for weight loss was common in early writings on anorexia nervosa and is still an important theme in some cases.

The present findings showed that students who fast had lower scores than those who do not fast on interpersonal alienation. The majority of subjects were Christians, representing also the major religion in Romania. Fasting is respected in general by the religious persons. Meaning that subjects are closed to religious rules, are used to going to church and fulfilling dietary restrictions during festal times. The result that students who fast are presenting less personal alienation is in congruency with the majority of studies, showing that the practice of religion has positive effects on personal, familial, social life, and on health [45,46,47]. Some recent study showed that a high prevalence of religious practice was associated with overweight/obesity, especially among Christian women [48].

Results proved that students who used to eat during nights obtained higher scores on personal alienation, so they had a low level of self-esteem. These findings are in congruence with previous researches on night-eaters. Individuals who used to eat during nights had higher scores on depression and lower self-esteem and usually eat later in the morning and present sleeping disturbance [49]. Further research would be interesting to focus on night-eating syndrome, in order to identify this psycho-pathological problem among students.

### Strength and Limitations of the Study

The strong points of the research are due to the fact that no study was lead before in pharmacy students in Romania and there are not many international studies focusing on this population.

The first limitation is due to the fact that pharmacy students were recruited from one single university, so results cannot be generalized for all pharmacy student population in Romania. The university is gathering persons from the north-eastern part of the country. Because some studies on this population found differences considering the native region of the participants, further researches could focus on it. The second limitation refers to the small number of male respondents, so a comparative analysis must be considered with precociousness. The third limitation is related to the fact that no psychological comorbidity was taken into consideration, like the presence of depressive syndrome or any psychiatric diseases. These factors could influence dietary habits or health-related behaviors (preferences for specific foods, odd preferences, compulsory eating, self-rewarding eating behaviors, etc.). For example, insomnia was associated with higher severity of disordered eating and studies showed that both insomnia and disordered eating symptoms were related to depression [50].

## 5. Conclusions

The results of the present study showed that eating disorders were identified having higher rates among pharmacy students than in general population in Romania. Considering some key factors like age, environment, fasting, sleeping, respecting main meals, or encouraging the consumption of fruits and vegetables could help students and university policy makers to promote healthier eating behaviors among pharmacy students.

## Figures and Tables

**Table 1:** Socio-demographic, anthropometric, and medical data.

Variables	Results ^1^
Gender	
female	83 (91.21%)
male	8 (8.79%)
Environment	
urban	54 (53.94%)
rural	37 (40.66%)
Religion	
Orthodox	70 (77.78%)
Catholic	10 (11%)
Others (Pentecostal, Adventist, etc.)	11 (11.21%)
Respecting religious fasts	29 (31.87%)
Weight (kg)	60.2 ± 10.56 (42 to 105)
Body mass index (BMI)	±
underweight	13 (14.3%)
normal weight	63 (69.2%)
pre-obese	12 (13.2%)
obese	0
Having a chronic disease	13 (14.29%)
Being under a medical treatment	15 (16.49%)

^1^ Number (N) and percent (%), Mean (M) and standard deviation (st.dev).

**Table 2:** Comparative results for EDI-3 subscales for men and women.

EDI-3 Subscales	Pharmacy Students	Pharmacy Students	General Population	General Population
	Male	Female	Male	Female
Drive for thinness	3.37 ± 4.30	9.26 ± 7.65	2.66 ± 3.66	8.03 ± 7.54
Bulimia	1.12 ± 1.80	4.87 ± 5.97	2.04 ± 2.38	2.00 ± 3.18
Body dissatisfaction	7.25 ± 6.62	17.03 ± 10.62	5.18 ± 6.13	10.29 ± 9.28
Low self-esteem	2.75 ± 2.37	8.08 ± 5.98	3.28 ± 4.85	2.97 ± 3.19
Personal alienation	6.62 ± 1.40	10.40 ± 4.48	3.55 ± 4.12	4.48 ± 3.45
Interpersonal insecurity	6.35 ± 5.75	13.81 ± 7.75	7.07 ± 5.87	6.73 ± 4.95
Interpersonal alienation	5.03 ± 6.34	11.98 ± 9.62	7.04 ± 4.48	7.13 ± 4.06
Interoceptive deficits	7.82 ± 2.72	12.20 ± 3.50	4.79 ± 5.16	6.10 ± 4.34
Emotional dysregulation	6.24 ± 1.88	7.74 ± 2.12	5.36 ± 5.83	6.03 ± 4.60
Perfectionism	4.68 ± 7.84	10.96 ± 10.87	11.79 ± 5.34	10.42 ± 5.42
Ascetism	5.19 ± 3.29	7.77 ± 4.00	6.41 ± 3.66	6.81 ± 3.82
Maturity fears	6.65 ± 5.28	17.97 ± 10.37	10.62 ± 4.07	13.23 ± 5.81

^1^ Means (M) and standard deviations (st.dev).

**Table 3:** Meals (breakfasts, lunches, dinners) per week.

How Many Times Per Week Do You Eat…	Never	1	2	3	4	5	6	Daily	M ± st.dev
breakfast	7.69%	5.49%	8.79%	16.48%	4.40%	14.29%	9.89%	32.97%	4.51 ± 2.34
lunch	1.10%	0%	2.20%	4.40%	12.09%	14.29%	6.59%	59.34%	5.92 ± 1.54
dinner	1.10%	0%	4.40%	9.89%	4.40%	13.19%	2.20%	64.84%	5.89 ± 1.72

^1^ number of meals (%).
